# Curvelet-Based Stochastic Noise Suppression for Downhole DAS Microseismic Data

**DOI:** 10.3390/s26175598

**Published:** 2026-09-03

**Authors:** Youyuan Zhang, Zhanguo Chen, Hao Chen, Leilei Cheng, Jian Dong, Zhixiang Wu, Zizheng Li

**Affiliations:** 1 SINOPEC Geophysical Research Institute Co., Ltd., Nanjing 211103, China; 2 Nanjing Branch, National Technology Innovation Center for Oil & Gas Geophysical Exploration, Nanjing 211103, China

**Keywords:** DAS, curvelet-transform, microseismic, stochastic noise, threshold

## Abstract

**Highlights:**

This study develops a curvelet-transform denoising framework for downhole distributed acoustic sensing (DAS) microseismic data and systematically evaluates four default thresholding strategies on synthetic and field records, recommending a robust, reference-window-free default for routine microseismic monitoring.

**What are the main findings?**

- Curvelet-domain thresholding suppresses stochastic DAS noise, recovering weak P-, S-, and reflected microseismic arrivals on field records with negligible spurious events.
- Among four default threshold strategies, MAD, quiet-window, and ECDF-percentile work out of the box; the knee-point default (β=0.2) used by the DASpy toolbox fails because its threshold sits inside the noise body.

**What are the implications of the main findings?**

- MAD per-sub-band soft thresholding is recommended as a robust default for DAS microseismic denoising, as it needs no blank reference window and is robust to non-stationary field noise.
- Curvelet denoising is mechanistically suited to the curved, direction-sparse wavefronts of downhole DAS records, offering a physically interpretable, no-tuning alternative to wavelet and Goldstein FK filtering.

**Abstract:**

Distributed acoustic sensing (DAS) converts fiber cables into dense strain-rate sensor arrays, capturing direct, reflected and guided seismic phases with ultra-fine spatial sampling. However, DAS interrogators suffer far stronger random noise than traditional geophones, limiting its microseismic imaging capacity. This work adopts curvelet-transform denoising to suppress noise. Curvelets partition the frequency–wavenumber plane into multiscale directional sectors; coherent wave energy concentrates in limited angular wedges, while stochastic noise disperses evenly across all transform coefficients, enabling noise-signal separation via wedge-wise thresholding. We test four default threshold schemes on synthetic downhole DAS microseismic data. Parameter tuning proves all methods deliver comparable performance, so we compare their out-of-box reliability for shale reservoir monitoring. Three noise-statistic-based strategies perform stably: median-absolute-deviation (MAD), quiet-window and empirical-cumulative-distribution-Function (ECDF percentile) thresholding. By contrast, the default knee-point algorithm from mainstream DAS toolboxes fails, as its preset threshold falls within noise components and barely removes interference. We propose MAD as a robust default for the tested downhole DAS microseismic setting for it estimates thresholds directly from noisy traces without blank reference windows and offers superior operational stability. Applied to field DAS records from a southwest China shale-gas horizontal monitor well, the MAD curvelet workflow greatly enhances microseismic arrivals with negligible spurious events. Benchmarks against standard 2D Daubechies-4 wavelet and adaptive Goldstein FK filtering verify curvelet denoising as a physically interpretable, efficient tool for DAS wavefields.

## 1. Introduction

Microseismic monitoring is a standard tool for evaluating hydraulic fracturing operations in unconventional reservoirs. The locations, magnitudes, and source mechanisms of induced microseismic events constrain estimates of fracture geometry, stimulated reservoir volume, and the spatio-temporal evolution of the fracture network [1,2]. Distributed acoustic sensing (DAS) has been increasingly adopted for microseismic monitoring because it repurposes a standard fiber-optic cable as a dense array of strain-rate sensors, with spatial sampling below 1 m, aperture extending over kilometers, and permanent installation behind casing that enables continuous monitoring without well intervention [3,4].

Beyond locating events and recovering source mechanisms, the high spatial sampling and wide aperture of DAS capture a richer wavefield than sparse geophone arrays. In addition to direct P and S arrivals, DAS records reflected waves from induced fractures and faults [5,6,7] and dispersive guided waves trapped in low-velocity reservoir layers [8,9]. Exploiting these phases, however, requires them to be recovered from the noisy DAS background in the first place. The same high spatial and temporal sampling that makes DAS attractive also creates a data-volume challenge. For example, a single downhole fiber recording continuously at 1 kHz with 1 m channel spacing generates several terabytes per day, motivating sparse representations that capture the essential wavefield information in far fewer numbers than the raw sample count.

DAS noise originates from multiple sources, including interrogator electronics, fiber heterogeneity, and borehole coupling. Among these, the stochastic component dominated by instrument noise can be substantially higher than that of conventional broadband seismometers and is the principal factor limiting weak-event detection. For microseismic events, which are inherently weak and often recorded near the detection threshold, this noise masks or distorts arrivals and limits the accuracy of downstream products such as missed detections in event catalogs, biased travel-time picks, and degraded moment tensor solutions. Other disturbances such as common-mode perturbations and transient optical spikes also occur in practice and are routinely handled by median filtering as a pre-processing step [10,11]. This study focuses exclusively on the stochastic noise component and on sparse transform-domain thresholding as the suppression strategy.

Existing approaches to stochastic noise suppression in DAS data span a range of methods. Early work treated the data trace-by-trace: wavelet thresholding [12], empirical mode decomposition [13], and median filtering each reduce the incoherent background along individual channels. While effective at reducing uncorrelated noise, these trace-by-trace methods ignore the spatial continuity that distinguishes signal from noise in DAS record sections. Some subsequent 2D approaches that exploit spatial coherence attempt to break this limitation, such as 2D edge detection with Sobel operators [14], adaptive bilateral filtering [15], principal component analysis [16], and self-supervised deep learning [17].

Several recent developments form the immediate background of this study. Qin et al. (2017) [18] introduced curvelet-domain thresholding to distributed vibration sensing, showing that the curvelet frame sparsely represents the 2D backscatter image and that thresholding its coefficients suppresses random noise while recovering the vibration signature. Although conducted on laboratory ϕ-OTDR data rather than seismic wavefields, this work first demonstrated curvelet sparsity for vibration sensing. Atterholt et al. (2021) [10] extended curvelet processing to surface DAS earthquake data, building a unified framework in which soft thresholding suppresses stochastic noise and velocity-dependent wedge muting removes coherent traffic noise. Isken et al. (2022) [11] took a complementary route in the frequency–wavenumber (FK) domain; they adapted the Goldstein InSAR filter, which multiplies the 2D FK amplitude spectrum by a scaled version of itself to enhance locally coherent wavefield components, and showed that it efficiently suppresses incoherent noise on borehole DAS recordings of active and passive sources. Most recently, Feng et al. (2025) [19] applied an empirical curvelet transform to 1D microseismic data, proposing an adaptive threshold strategy combined with an improved non-local means filter. Their work, however, used conventional geophone traces rather than DAS, and focused on developing a new hybrid denoiser rather than on systematically comparing threshold strategies or addressing the 2D curved-wavefront geometry that characterizes DAS record sections.

The high spatial–temporal sampling of downhole DAS data, together with the curved-wavefront geometry of microseismic arrivals, motivates the adoption of a representation that is both directionally selective and multiscale. Directional selectivity enables separation of P- and S-wave energy at different apparent velocities, while multiscale characteristics capture the sharp onset and rapid decay of microseismic pulses. These desirable features are intrinsic to the curvelet frame. Polar FK-domain tiling yields directional selectivity, anisotropic scaling delivers multiscale sharpness, and the tight-frame property guarantees energy conservation. This constitutes a favorable attribute for thresholding workflows, since coefficient shrinkage preserves total energy in expectation.

Threshold selection is decisive in curvelet-domain denoising. An ill-chosen threshold can either leave too much noise or erase genuine signal. Four candidate strategies have emerged in the literature. The first, Donoho and Johnstone (1994) [20] and Starck et al. (2002) [21], introduced per-sub-band median-absolute-deviation (MAD) soft thresholding. This method estimates the noise standard deviation directly from the curvelet coefficients within each wedge of the noisy data, requiring no separate noise reference window. The second strategy, proposed by [10], estimates thresholds from a quiet reference window (abbreviated here as quiet-window). The user selects a time segment of the record section that is largely free of coherent signal; the curvelet coefficients of this segment serve as a pure-noise sample, and per-sub-band thresholds are derived from their distribution. The third strategy, introduced by [22], is the empirical cumulative distribution function approach (abbreviated here as ECDF-percentile). Like the quiet-window method, it uses a noise reference segment, but it makes no distributional assumption about the coefficients. The threshold is simply the P-th percentile of the empirical cumulative distribution function of the coefficient amplitudes taken from the quiet window. The fourth candidate is the knee-point strategy. Its algorithmic foundation comes from [23], who developed a general-purpose knee-detection procedure (Kneedle) for locating inflection points in discrete cumulative distribution curves within distributed computing systems. Hu and Li (2024) [24] subsequently incorporated it into their open-source DASPy toolbox as an alternative thresholding path for curvelet denoising.

In shale reservoirs, DAS microseismic recordings exhibit strong sparsity in the curvelet domain; the bulk of the energy is concentrated in a small fraction of the coefficients (as shown later). This study applies curvelet-domain thresholding to downhole DAS microseismic data from a horizontal monitoring well in a shale gas field in southwest China. We pursue two main aims. First, we assess the practical performance of the four thresholding strategies—MAD, quiet-window, ECDF-percentile, and knee-point—at their default parameter settings, then identify and correct underperforming defaults so that each method can deliver effective denoising without per-dataset tuning. This is a benchmarking exercise on an established technique, not a ranking or a new method. Once appropriately re-tuned, all four strategies achieve comparable noise suppression. Second, we provide an explicit interpretation of the geometric match between curvelet frames and borehole DAS wavefields. The anisotropic directional decomposition naturally matches the curved, direction-sparse features of two-dimensional downhole DAS records. This specific scenario has received little attention within existing curvelet-denoising studies targeting laboratory-OTDR, surface-deployed DAS, and single-channel geophone datasets.

In addition to comparing the four thresholding variants, we benchmark curvelet denoising against two reference methods—2D wavelet (Daubechies-4) thresholding and the adaptive Goldstein FK filter [11]. All methods adopt their standard configurations and are tested on the same field dataset. The results demonstrate that curvelet denoising can serve as a viable alternative for attenuating random noise within continuous downhole DAS records.

## 2. Methodology

### 2.1. Curvelet Transform Fundamentals

The key observation underlying this study is the contrast in how coherent signal and incoherent stochastic noise populate the frequency–wavenumber (FK) plane. A microseismic wavefront is coherent in the FK sense. It follows a curved trajectory in the time–channel record section and therefore occupies a specific, directionally localized region of the FK plane, for instance, P-wave energy along a high-apparent-velocity ray and S-wave energy along a moderate-apparent-velocity ray. Stochastic interrogator noise, by contrast, has no preferred direction and spreads its energy roughly uniformly across the FK plane. A representation that partitions the FK plane into oriented, multiscale cells therefore concentrates coherent signal energy into a few direction-specific cells while leaving noise spread thinly everywhere. Per-cell coefficient thresholding then separates coherent signal from incoherent noise, which is the central insight behind curvelet denoising.

Candès and Donoho (2004) [25] construct curvelets as a family of waveforms whose FK-domain envelopes tile the plane in a polar grid. Denote the 2D Fourier coordinates by $\omega =(\omega_{x},\omega_{t})$—wavenumber and frequency—and their polar representation by radial coordinate ρ=∥ω∥ and azimuth $\varphi =arctan(\omega_{t}/\omega_{x})$. The FK plane is partitioned into concentric dyadic annuli indexed by scale j and, within each annulus, into equi-spaced angular sectors indexed by orientation l. The frequency-domain envelope of the curvelet at scale j and orientation l is the separable window given in Equation (1):

(1) $$\Psi_{j,l}(\rho ,\varphi )=2^{-3j/4}\;H(2^{-j}\rho )\;A\left(\frac{\;2^{\left\lfloor j/2\right\rfloor }\;\varphi }{2\pi }\right).$$

where the radial profile H(ρ) is a smooth bump supported on ρ∈[π,2π] and the angular profile A(φ) is a smooth window on ϕ∈[−π,π]. The scale-dependent angular factor $2^{\left\lfloor j/2\right\rfloor }$ enforces the parabolic scaling relation that defines the transform: at scale j, a curvelet’s effective FK-domain support obeys

(2) $$\mathrm{width}\propto 2^{-j},\;\;\mathrm{length}\propto 2^{-j/2},\;\;\Rightarrow \;\;\mathrm{width}\propto (\mathrm{length}{)}^{2}.$$

**Figure 1:**
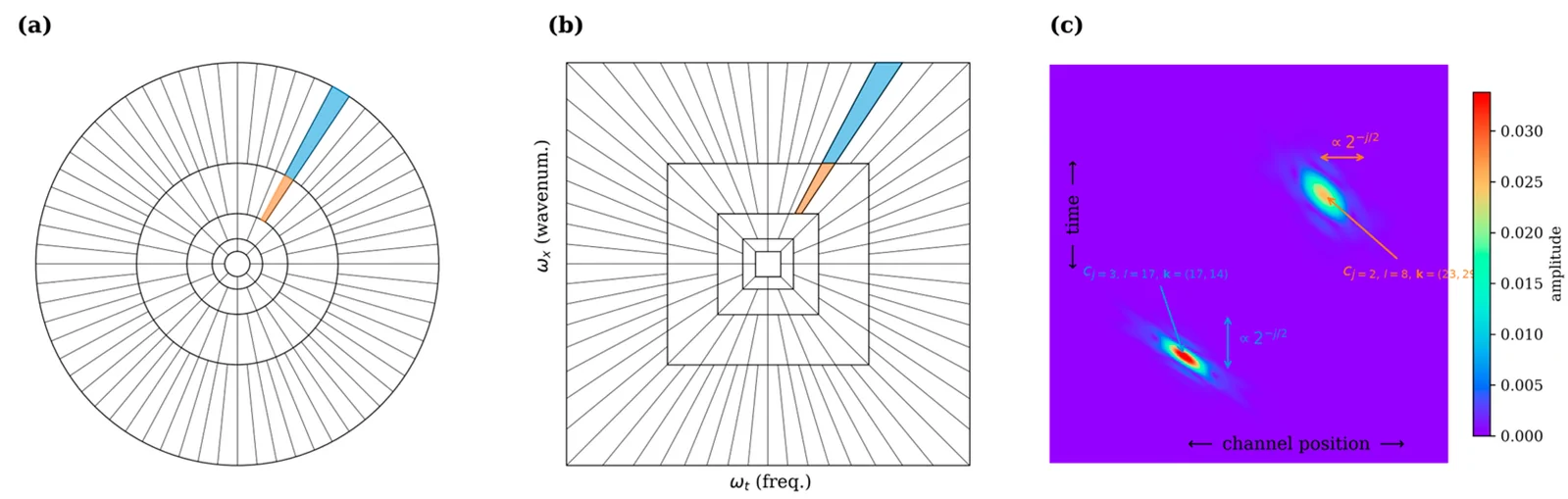
Curvelet transform principle. (**a**) Conceptual polar tiling of the FK domain. (**b**) Digital implementation on the Cartesian FFT grid via the wrapping algorithm. (**c**) Spatial-domain curvelet atoms obtained by inverting a single impulse coefficient from each highlighted FK wedge.

Equation (2) is the property that distinguishes curvelets from both wavelets (which are isotropic) and ridgelets (which are directional but lack multiscale anisotropy). At fine scales (j large), the support becomes extremely elongated—a long, thin wedge aligned with the local orientation—enabling the curvelet to adapt to the geometry of a curved wavefront; it matches the wavefront’s smooth tangent direction over a long extent while resolving its sharp transverse profile with a narrow cross-section. At coarse scales (j small), the support is squatter and the curvelet approaches an isotropic blob. This anisotropic scaling is illustrated in Figure 1a.

Equation (1) describes a polar partition, but digital images are sampled on a Cartesian grid and their 2D FFT is indexed by rectangular coordinates. Implementing the polar tiling directly would require interpolating the Cartesian FFT samples onto a polar grid, which introduces resampling errors and breaks the exact reconstruction property. The Fast Discrete Curvelet Transform (FDCT) [26] circumvents this problem through the wrapping algorithm:

(1) Compute the 2D FFT of the N×N input image on the standard Cartesian grid.
(2) For each scale j and orientation l, multiply the FFT array by the Cartesian realization of the window $\Psi_{j,l}$ (Figure 1b). On the Cartesian grid, each polar wedge becomes a parallelogram—the Cartesian shear encodes the wedge’s orientation.
(3) Rather than interpolating this parallelogram onto a rectangular sub-band, the wrapping operation re-indexes the FFT samples via modulo arithmetic, collecting the parallelogram’s content into a rectangle centered at the origin. This is a lossless rearrangement of the existing FFT samples: no interpolation, no resampling.
(4) An inverse FFT of each wrapped rectangle yields the curvelet coefficients $c_{j,l,k}$ for that wedge, where $k=(k_{1},k_{2})$ indexes the spatial position of the coefficient.

The wrapping algorithm has two practical consequences. First, because it only re-indexes existing FFT samples, the transform is numerically exact; forward FDCT followed by inverse FDCT recovers the input image to within floating-point precision. Second, the spatial-domain curvelet atom obtained by inverting a single coefficient (Figure 1c) is a localized, oriented waveform obeying the parabolic scaling (Equation (2)). Its major axis is aligned with the wavefront direction, which is rotated by 90° relative to the FK-domain wedge orientation, because the FK wedge points along the propagation direction (the wavefront normal), while the spatial atom extends along the wavefront itself. This is a general property of the Fourier transform: a line-like feature in the spatial domain maps to a perpendicular line in the frequency domain.

### 2.2. Denoising Framework

We adopt the standard sparse thresholding paradigm:

(1) Forward FDCT: Compute curvelet coefficients $c_{j,k,l}$ of the noisy DAS record section d(x,t), where j indexes scale, l indexes orientation (wedge), and k indexes spatial position within the wedge.
(2) Threshold: Apply a wedge-dependent threshold $\tau_{j,l}$ to shrink or zero coefficients, producing modified coefficients ${\tilde{c}}_{j,k,l}$.
(3) Inverse FDCT: Reconstruct the denoised record section $\tilde{d}(x,t)$.

We adopt an additive, spatially uncorrelated noise model given in Equation (3):

(3) $$d(x,t)=s(x,t)+n(x,t),\;\;n∼N(0,\sigma^{2}).\;$$

where s is the noise-free signal. This is consistent with the dominant interrogator noise source [27]. Non-stochastic disturbances (common-mode perturbations and transient spikes due to optical fading) are assumed to have been removed by the pre-processing median filter and do not enter the denoising comparison.

For the shrinkage function (step 2 above), we use soft thresholding throughout. Given a threshold λ, soft thresholding shrinks each coefficient toward zero by λ given in Equation (4):

(4) $${\tilde{c}}_{j,k,l}=sign(c_{j,k,l})\cdot max(|c_{j,k,l}|-\tau_{j,l},\;0).$$

**Figure A1:**
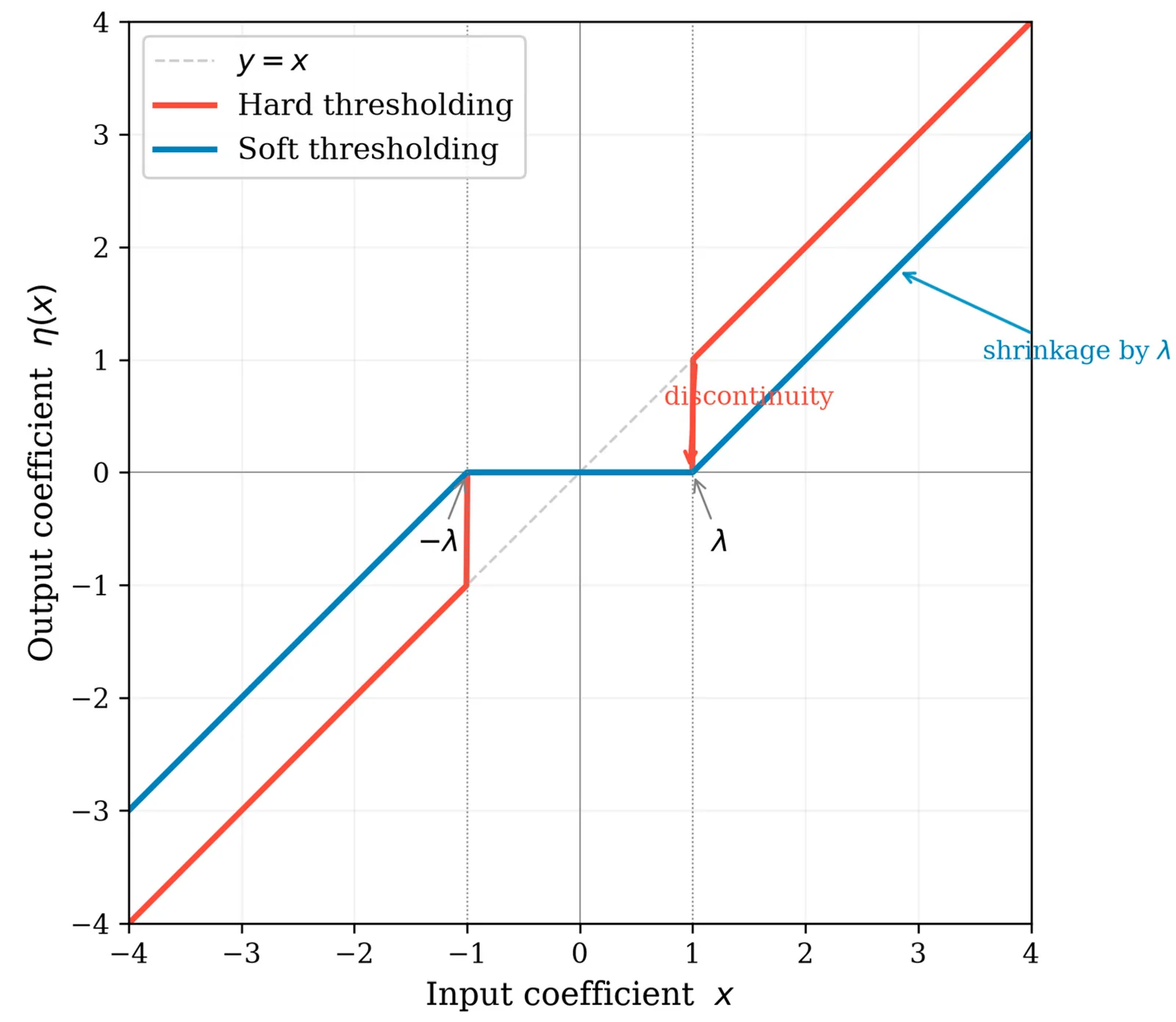
Hard versus soft thresholding transfer curves. Hard thresholding (red) preserves coefficients above |λ| unchanged and zeros the rest; the step at ±λ (arrow) is the discontinuity responsible for ringing artifacts after inverse transformation. Soft thresholding (blue) is continuous but shrinks all coefficients by λ, visible as the offset between the dashed identity line and the soft thresholding curve for |x|>λ.

We select soft thresholding over hard thresholding—which zeros sub-threshold coefficients but leaves supra-threshold ones unchanged—because soft thresholding is continuous and avoids the ringing artifacts that hard thresholding’s discontinuity can introduce along wavefront edges [20,21]. The hard thresholding formula and a visual comparison of the two operators are provided in the Appendix A (Figure A1). The threshold $\tau_{j,l}$ depends on scale j and orientation l but is uniform across spatial positions k within a wedge, reflecting the assumption of a spatially stationary noise floor. The strategy for estimating $\tau_{j,l}$ is the subject of the next section.

### 2.3. Threshold Strategies

Strategy 1: MAD per-sub-band soft thresholding [20,21]. This is our recommended strategy.

The threshold is computed directly from the noisy data. For each wedge (j,l), we estimate the noise standard deviation via the median absolute deviation, scaled for Gaussian consistency given in Equations (5) and (6):

(5) $$\sigma_{j,l}=\frac{{MAD}_{j,l}}{0.6745},\;\;{MAD}_{j,l}=median\;\left(\left|c_{j,k,l}-median(c_{j,k,l})\right|\right).$$

(6) $$\tau_{j,l}^{\left(MAD\right)}=k\cdot \sigma_{j,l}.$$

where k is a user-specified multiplier and the factor 0.6745 converts MAD to the standard deviation of a Gaussian distribution, $\sigma_{j,l}$. We use k=2.0, a commonly used heuristic multiplier for soft thresholding in curvelet and wavelet denoising [21]; it is distinct from the universal threshold $\sigma_{j,l}\;\sqrt{2lnN}$ of [20], where N denotes the number of curvelet coefficients in the current wedge. We employ Equation (5)—in its level-dependent form—for the wavelet VisuShrink baseline in the Discussion.

Strategy 2: Quiet-window-based threshold [10].

Atterholt et al. (2021) [10] estimate thresholds from a quiet time window selected from the DAS data. The procedure is: (a) select a segment of the record section that contains minimal coherent signal, (b) compute the FDCT of this noise-only segment, (c) characterize the coefficient amplitude distribution within each wedge, and (d) use these distributions to determine $\tau_{j,l}$. The original paper describes the threshold as derived from the per-wedge coefficient amplitude distribution without specifying a particular estimator; we implement it as the per-wedge noise standard deviation multiplied by the same factor k=2.0 for fair comparison with MAD.

Strategy 3: ECDF-percentile threshold [22].

Yang et al. (2020) [22] proposed this strategy for continuous wavelet transform processing of seismic ambient noise. The threshold is set to the P*-th* percentile of the coefficient amplitude distribution estimated from a signal-free segment given in Equation (7):

(7) $$\tau_{j,l}^{\left(ECDF\right)}={ECDF}_{j,l}^{-1}(P).$$

where ${ECDF}_{j,l}$ is the empirical cumulative distribution function of $\left|c_{j,k,l}\right|$ from the quiet segment, and we set P=99%. This strategy requires a separate quiet window but makes no parametric assumption about the coefficient distribution shape.

Strategy 4: Knee-point threshold (a signal–noise-boundary method) [24].

The knee-point strategy is a boundary-detection thresholding method, and it attempts to locate the boundary between noise-dominated and signal-dominated coefficients directly from the noisy data. For a given wedge, sort the coefficient amplitudes in ascending order: $a_{1}\leq a_{2}\leq \cdots \leq a_{N}$. The empirical CDF at $a_{i}$ is $F_{i}=i/N$. To isolate the knee, a tilt correction is applied; the straight line connecting the endpoints $\left(a_{1},F_{1}\right)$ and $\left(a_{N},F_{N}\right)$ is subtracted from the data curve, yielding $a_{i}-s\cdot F_{i}$, where $s=\left(a_{N}-a_{1}\right)/\left(F_{N}-F_{1}\right)$. The index $i*=arg{min}_{i}\left(a_{i}-s\cdot ;F_{i}\right)$ marks the point of greatest departure from the straight line—the knee point and the corresponding amplitude $a_{i*}$ is the knee amplitude, an estimate of the signal-noise boundary. The final threshold τ is obtained by scaling the knee amplitude with a scale factor β:

(8) $$\tau =\beta \cdot a_{i*}$$

where β controls the position of the threshold relative to the detected boundary: β=1 places the threshold at the knee itself, while β<1 lowers it into the noise body and β>1 raises it into the signal tail. DASPy adopts β=0.2 as its default when no noise reference segment is supplied [24].

## 3. Synthetic Data Examples

### 3.1. DAS Microseismic Data Synthesis

Microseismic events induced during hydraulic fracturing are predominantly shear failures on pre-existing fractures [1,2,28]. Injected fluid raises pore pressure, reducing the effective normal stress on favorably oriented discontinuities until the resolved shear stress exceeds the Coulomb failure criterion. The resulting slip is parallel to the fracture plane, producing a double-couple (DC) source mechanism parameterized by strike $\phi_{s}$, dip δ, and rake λ [29]. We adopt a pure DC source with $\left(\phi_{s},\delta ,\lambda )=(0^{\circ },90^{\circ },90^{\circ }\right)$— a dip-slip shear rupture on a vertical fault plane striking along the fiber (the x*–*z plane); its moment-tensor eigenvalues (−1,0,+1) confirm a pure shear source with no isotropic component, consistent with a hydraulic-fracturing-induced shear event.

The forward model computes a DAS record section d(x,t)—strain rate as a function of channel position x and time t—as the superposition of direct P and S body waves via analytical ray theory in a homogeneous, isotropic elastic medium. A detailed description of the DAS record computation is provided in Appendix B.

**Figure 2:**
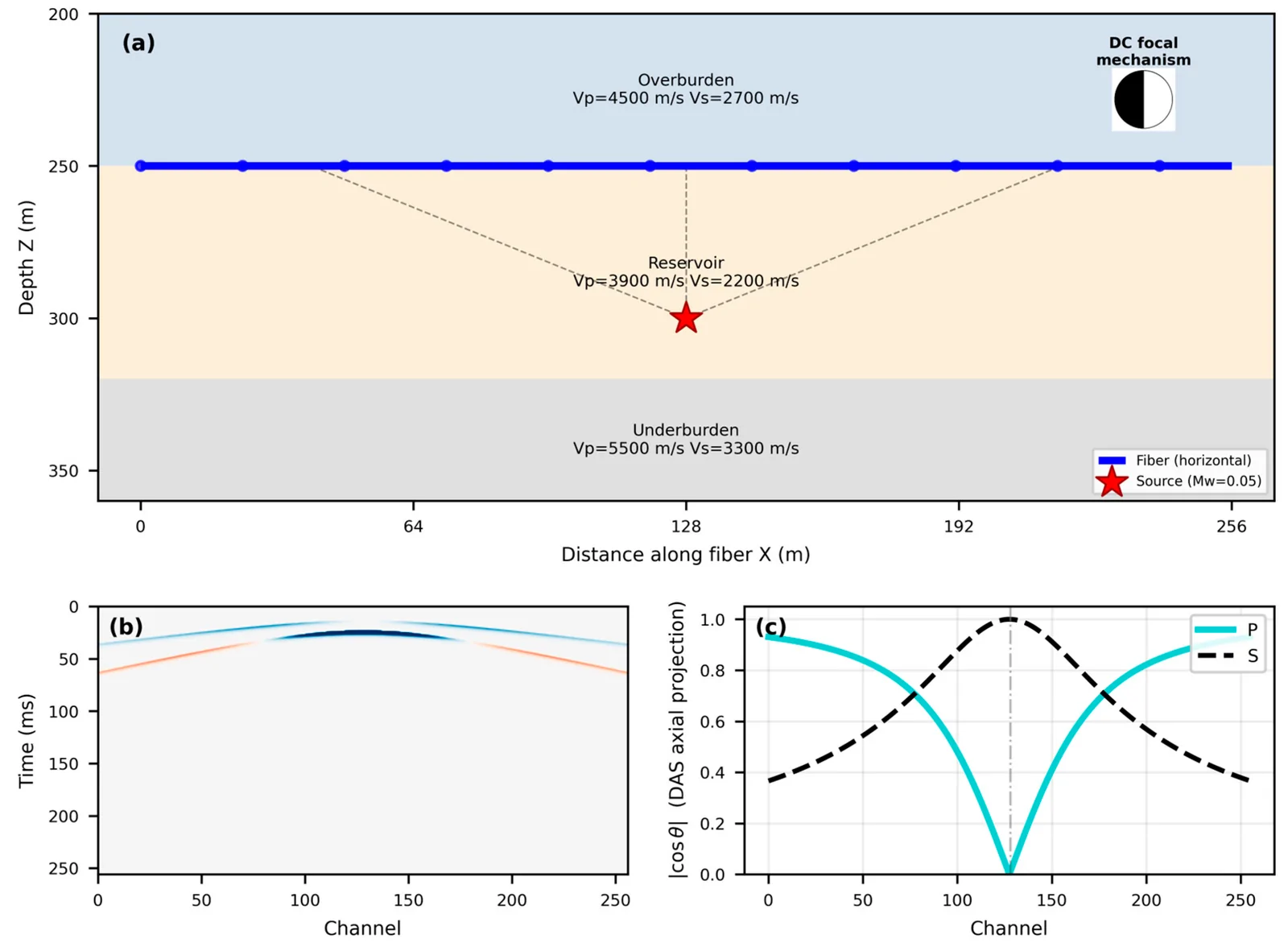
Synthetic data overview. (**a**) Observation geometry (2D vertical x–z cross-section). Inset: focal mechanism (beachball) for the dip-slip shear source (strike $0^{\circ }$, dip $90^{\circ }$, rake $90^{\circ }$). Dashed lines show example ray paths. (**b**) Noise-free synthetic DAS record. (**c**) DAS axial projection geometry: |cosθ| versus channel for P (cyan, solid) and S (black, dashed) polarizations.

In the synthetic example, the fiber is deployed as a horizontal section at depth z=250 m within a homogeneous formation with $v_{P}=3900$ m/s, $v_{S}=2200$ m/s, and ρ=2500 kg/m^3^, representing the in-reservoir horizontal monitoring section of a downhole DAS well. The 256 channels span x=0–255 m at 1 m spacing. The DC source ($M_{w}=0.05$, $f_{c}\approx 150$ Hz) is placed directly beneath the fiber center (50 m deeper) so that every source–receiver ray path lies in the vertical x*–*z plane, as shown in Figure 2a. The SH polarization ${\hat{p}}_{SH}=\hat{r}\times \hat{z}$ is then perpendicular to this plane (along $\hat{y}$) and hence orthogonal to the horizontal fiber ($\hat{x}$) at every channel, giving $|cos\theta_{SH}|\equiv 0$ and vanishing SH response everywhere. The record therefore contains only the direct P and SV arrivals.

Figure 2b displays the noise-free synthetic data. The S-wave (SV) arrival dominates the record section as a steeply curved event with an apparent velocity of approximately 2200 m/s, while the earlier P-wave arrival is clearly visible but weaker. The P-wave is suppressed relative to the S-wave by two compounding factors: (i) the strain-rate amplitude factor ($\propto 1/v^{2}$), which gives S an intrinsic $(v_{P}/v_{S}{)}^{2}\approx (3900/2200{)}^{2}\approx 3.1$ advantage, and (ii) the DAS axial projection—near the channel directly above the source the P-wave ray is nearly vertical (parallel to $\hat{z}$), so its particle motion is nearly orthogonal to the horizontal fiber ($|cos\theta_{P}|\ll 1$), whereas the SV particle motion has a large fiber-parallel component ($|cos\theta_{SV}|\to 1$), as shown in Figure 2c.

### 3.2. Threshold Strategy Comparison

We begin by comparing the four threshold strategies under a common FDCT configuration ($N_{scales}$= 5, $N_{angles}$ = 32). The noise-distribution-driven strategies are used at their standard defaults: *k* = 2.0 for MAD and quiet-window, P = 99% for ECDF-percentile, and β = 0.2 for the knee-point. Strategies (1) MAD and (4) knee-point operate directly on the noisy data; strategies (2) quiet-window and (3) ECDF-percentile are given an independent pure-noise reference window of identical dimensions, generated as an Additive White Gaussian Noise (AWGN) realization with the same standard deviation as the noise added to the signal. This design isolates differences in the estimation logic rather than in the input data.

**Figure 3:**
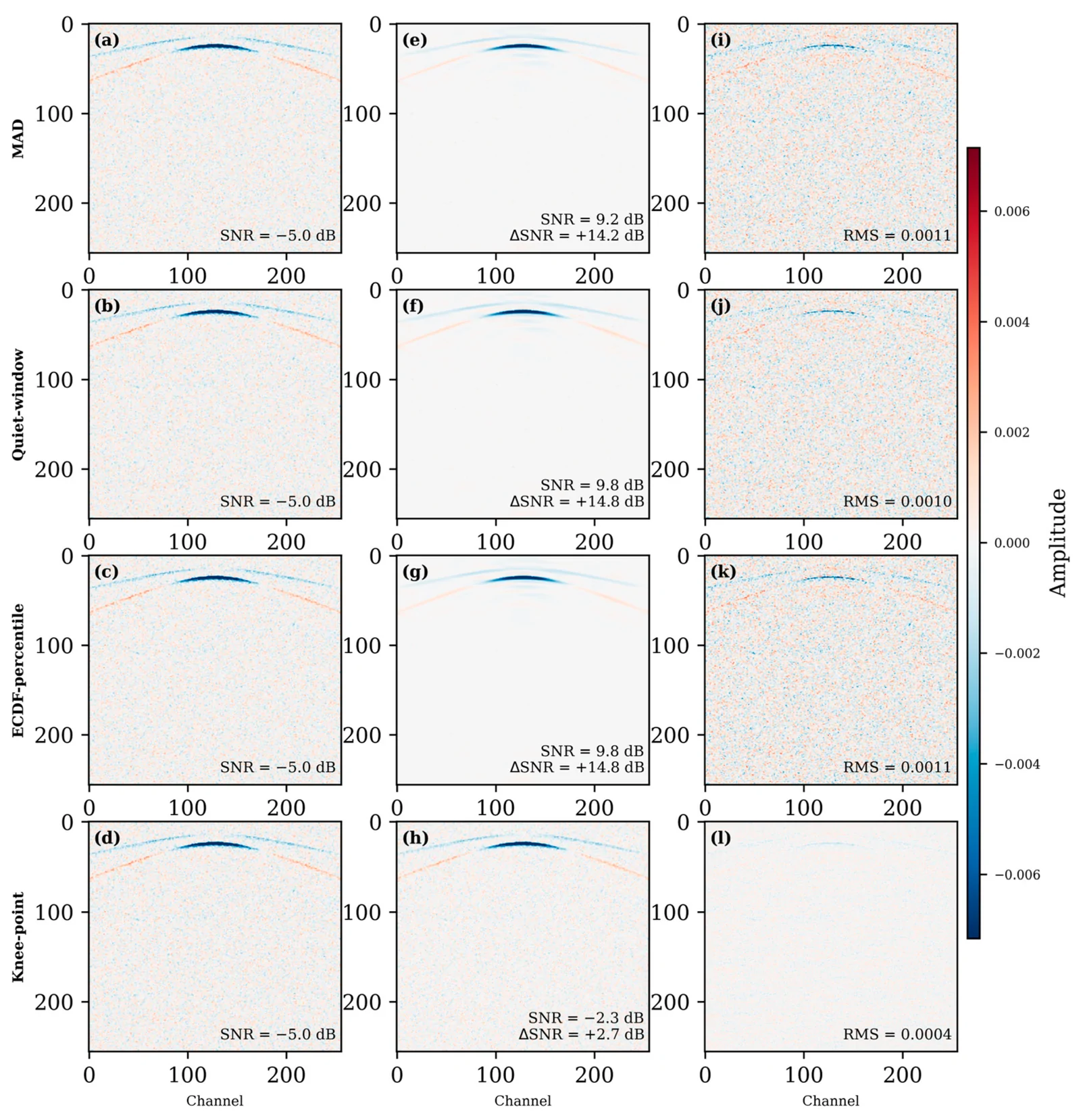
Comparison of thresholding strategies. (**a**–**d**) Noisy records with ${SNR}_{in}=-5\;dB$. (**e**,**i**) Denoised result and removed noise using MAD thresholding. (**f**,**j**) Same as (**e**,**f**) but for quiet-window thresholding. (**g**,**k**) Same as (**e**,**f**) but for ECDF-percentile thresholding. (**h**,**l**) Same as (**e**,**f**) but for knee-point thresholding.

Figure 3 displays the denoised record sections at the most challenging noise level (${SNR}_{in}=-5\;dB$), where the noise Root Mean Square (RMS) exceeds the signal RMS by a factor of ~1.8. Each row presents one strategy in a three-column layout: noisy input (left), denoised output (center), and residual—the difference between the two (right). This layout reveals both what each strategy preserves and what it removes. The three noise-distribution-driven strategies—MAD (row 1), quiet-window (row 2), and ECDF-percentile (row 3)—all perform well, delivering SNR improvements of 14.2 dB (Figure 3e), 14.8 dB (Figure 3f), and 14.8 dB (Figure 3g), respectively. The knee-point strategy (row 4), by contrast, yields a markedly poorer result: most of the noise persists and the SNR gain is only 2.7 dB.

These results indicate that, at their standard defaults—k=2.0 for MAD and quiet-window and P=99% for ECDF-percentile—the three noise-distribution-driven strategies are effective out of the box, whereas β=0.2 for the knee-point is not.

**Figure 4:**
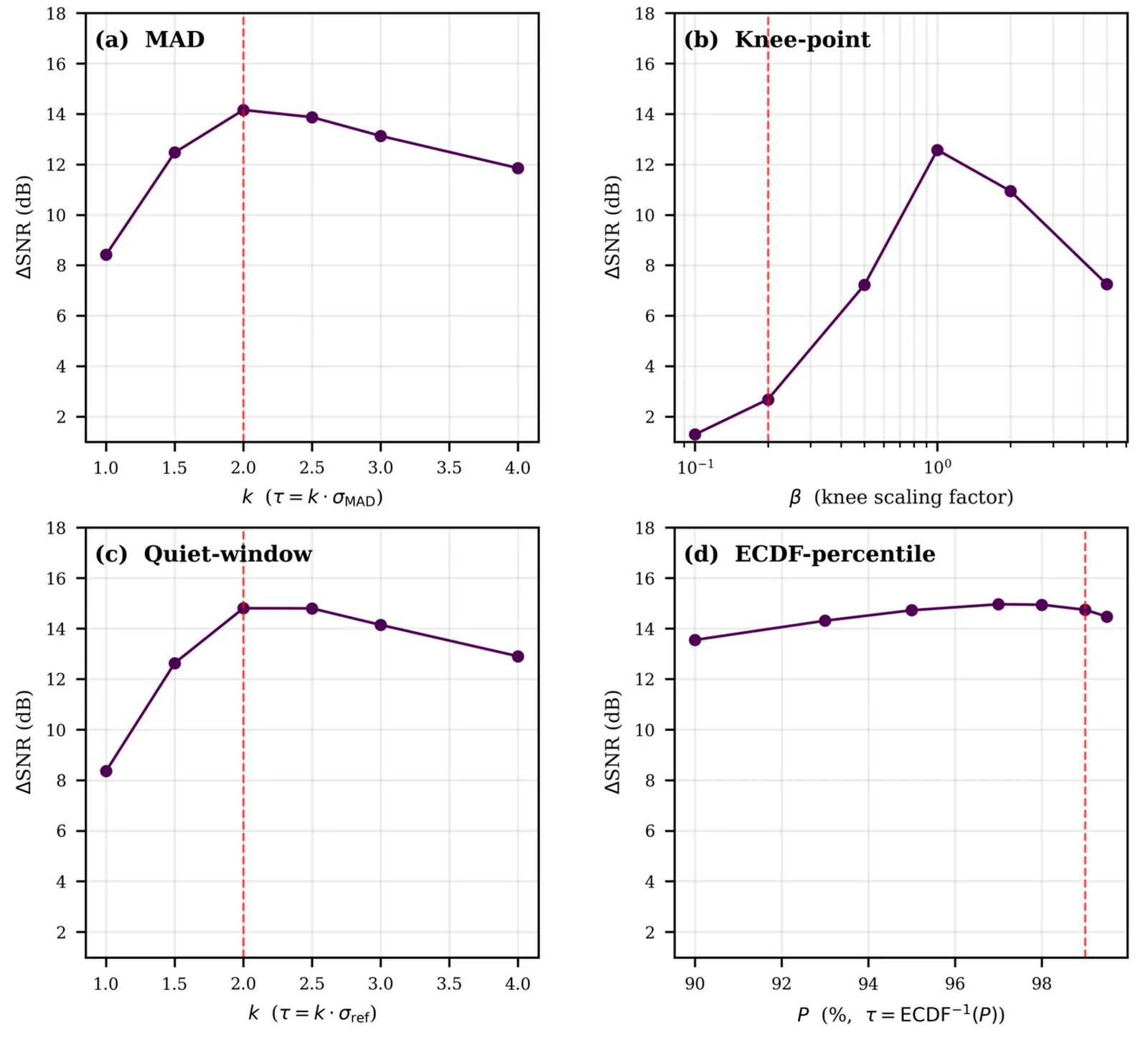
Parameter sweep results for the four thresholding strategies. (**a**) MAD thresholding as a function of the multiplier k. (**b**) Knee-point thresholding as a function of the scale factor β. (**c**) Quiet-window thresholding as a function of the multiplier k. (**d**) ECDF-percentile thresholding as a function of the percentile P. In each panel, the red dashed line indicates the default parameter value.

To determine whether the knee-point strategy’s poor showing reflects a fundamental limitation or merely an unsuitable default parameter, we conduct a systematic four-parameter sweep (Figure 4). For each strategy, we vary its controlling parameter over a broad range and track the resulting ΔSNR, seeking the configuration that maximizes denoising performance in the downhole DAS microseismic setting.

For MAD and quiet-window (Figure 4a,c), ΔSNR as a function of the multiplier k peaks close to the default k=2.0; the out-of-the-box setting is essentially optimal. For the knee-point strategy (Figure 4b), ΔSNR is near zero at the default β=0.2 and rises monotonically with β, reaching competitive values only at substantially larger scaling factors (β=1.0). This confirms that the poor performance seen in Figure 3 results from a misplaced default, not an intrinsic deficiency of the knee-detection logic. For ECDF-percentile (Figure 4d), ΔSNR is relatively flat across the tested range P=90–99.5%, and the default P=99% already yields strong denoising.

**Figure 5:**
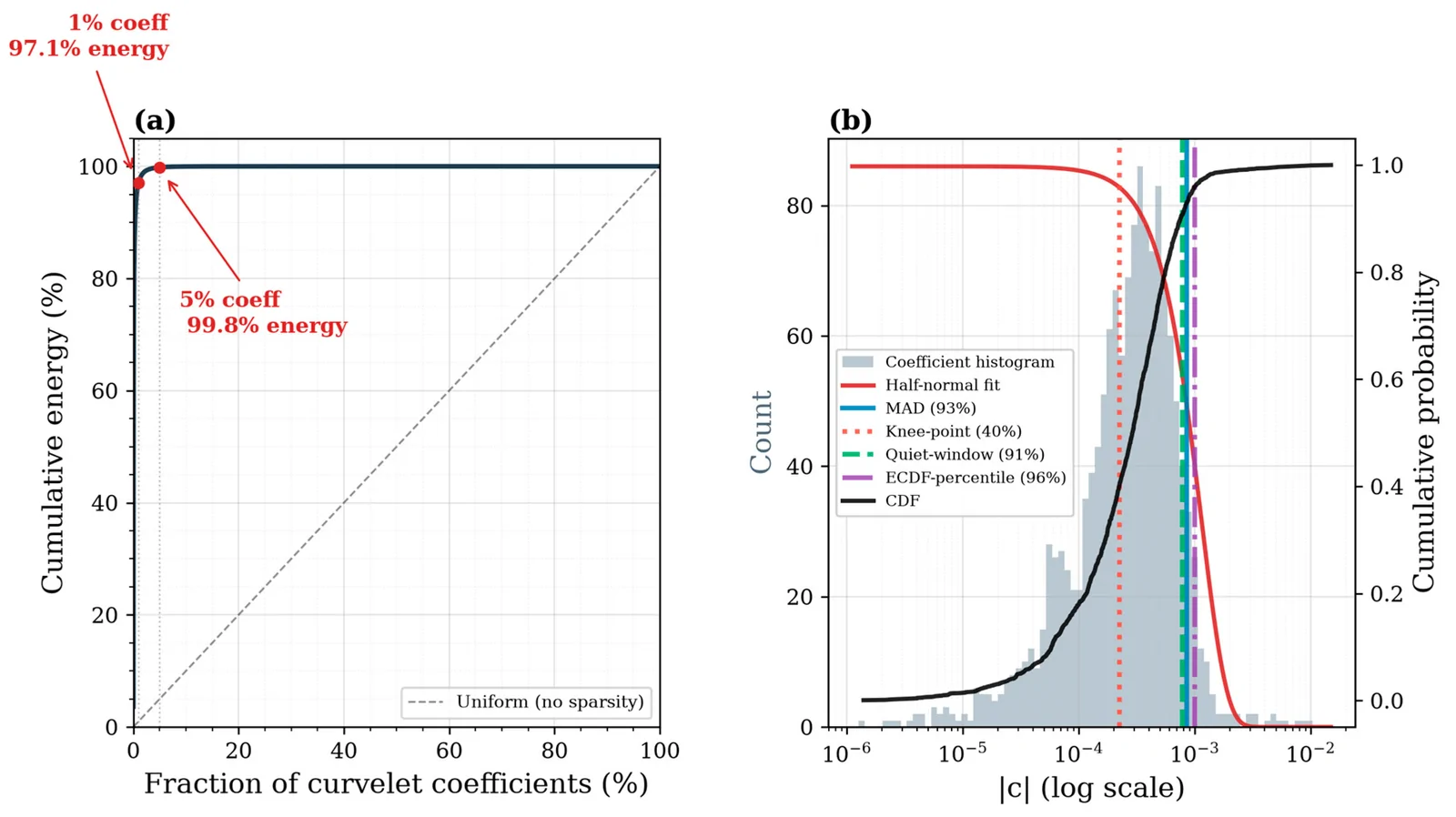
Sparsity and thresholding analysis of curvelet coefficients. (**a**) Lorenz curve of curvelet-coefficient energy for the clean synthetic data. (**b**) Coefficient-amplitude distribution for the most energetic wedge at scale 3 from noisy data, with half-normal fit, CDF, and default threshold positions of the four strategies.

The success of the noise-distribution-driven strategies at their default parameters can be understood from the sparsity structure of the signal. DAS microseismic recordings in shale reservoirs are dominated by direct P and S arrivals with hyperbolic moveout; in the curvelet domain, this wavefield collapses into a small number of high-amplitude coefficients, as quantified in Figure 5.

Figure 5a quantifies the energy concentration of the clean synthetic data in the curvelet domain. The horizontal axis gives the cumulative fraction of coefficients (sorted by decreasing magnitude); the vertical axis gives the corresponding cumulative fraction of total energy. The Lorenz curve hugs the upper-left corner; the top 1% of coefficients carry 97.1% of the total energy and the top 5% carry 99.8%. As a baseline, the diagonal dashed line represents a uniform energy distribution, for which 97% of the coefficients would be required to reach 97% of the energy. The degree to which the curve deviates from the diagonal is a direct measure of sparsity, and the microseismic signal is seen to be extremely sparse in this representation.

Figure 5b grounds this sparsity argument in the actual threshold placement. It shows the coefficient-amplitude distribution of the most energetic wedge at scale 3, extracted from noisy data. The gray histogram gives the empirical distribution of |c|; the red curve is a half-normal fit. The close agreement between the histogram body and the half-normal envelope validates the AWGN noise model in the curvelet domain. The black curve is the empirical CDF. Four colored vertical lines mark the default threshold τ of each strategy on this wedge. MAD (blue solid), quiet-window (green dashed), and ECDF (purple dash-dotted) all fall in the tail of the distribution, near the 91–96% quantile of the CDF; they zero only the extreme-noise coefficients while preserving the bulk of the signal. The knee-point threshold (red dotted, β = 0.2) sits near the 41st percentile of the CDF, deep within the main body of the noise distribution. At this setting, roughly 41% of coefficients are zeroed while nearly 60% of the noise coefficients survive. Together, Figure 4 and Figure 5 explain the observed contrast: MAD, quiet-window, and ECDF succeed at their defaults because their thresholds land in the noise tail; the knee-point strategy fails because its default β = 0.2 anchors the threshold inside the noise body.

In summary, MAD, quiet-window, and ECDF-percentile all position the threshold in the tail of the noise distribution at their default parameters and achieve effective curvelet-domain denoising. On field data, however, noise characteristics are non-stationary; a window that is quiet at one set of channels may contain coherent energy elsewhere, so a threshold derived from a single reference window does not generalize reliably across the full record section. Quiet-window and ECDF-percentile are therefore less convenient than MAD, which estimates the threshold directly from the noisy data with no reference window to select. On grounds of both simplicity and operational robustness, we recommend MAD thresholding as the preferred strategy for curvelet-domain denoising of DAS microseismic data in this study.

## 4. Field Data Application

To assess whether the curvelet denoising framework developed on synthetic data transfers to real DAS microseismic monitoring, we apply it to continuous recordings from a horizontal monitoring well in a shale gas field in southwest China.

### 4.1. Geological Setting and DAS Acquisition

**Figure 6:**
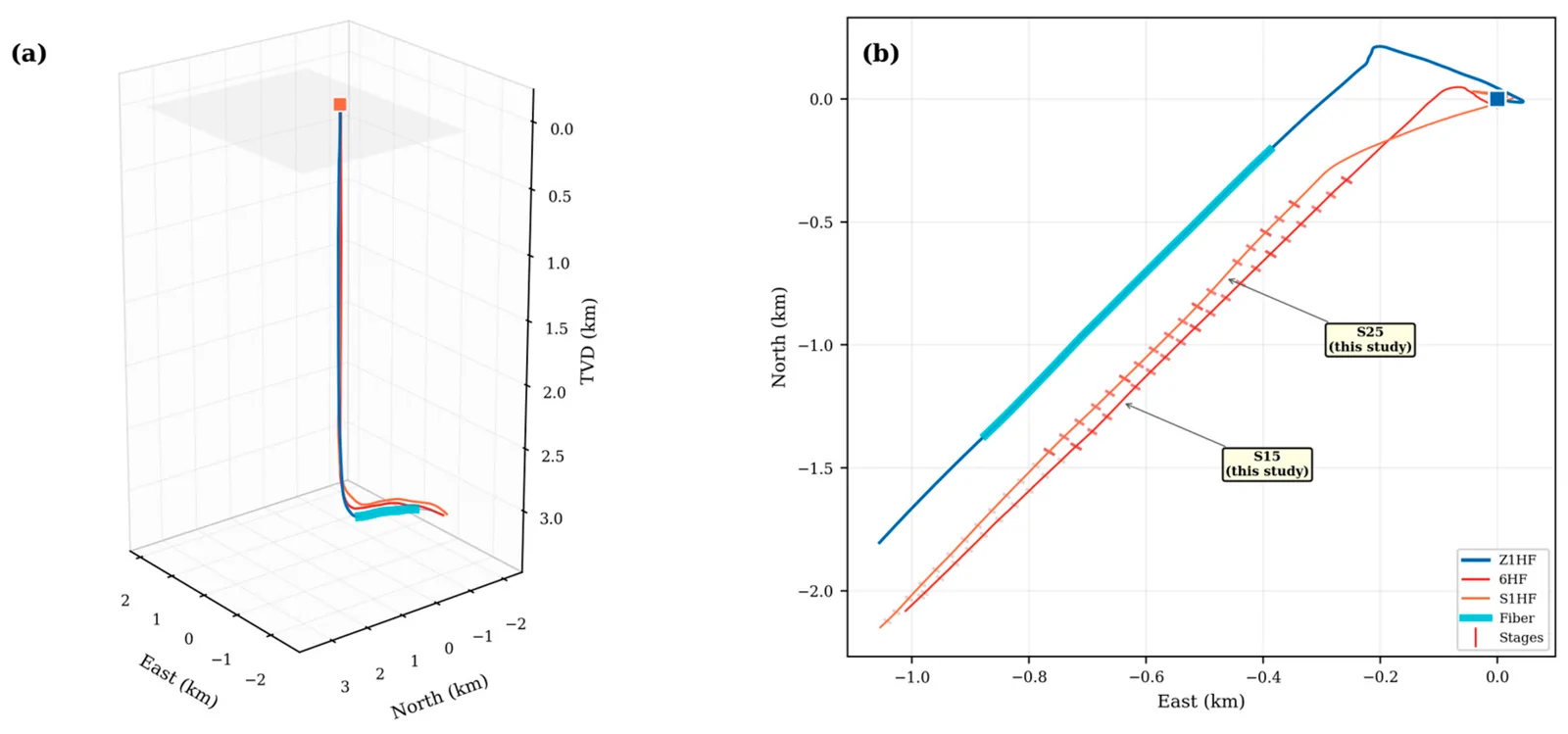
Layout of the three-well cluster in a shale gas field, southwest China. (**a**) 3D view of the well geometry. (**b**) Map view of the downhole locations.

The data come from a three-well cluster in a shale gas field in southwest China (Figure 6). The monitoring well Z1HF is completed with a single-mode fiber-optic cable permanently installed behind the production casing, protected by metal tubing and cemented in place. The fiber extends along the wellbore; the horizontal section within the target shale reservoir spans, highlighted in cyan in Figure 6a,b. Data were acquired continuously using a HiFi DAS interrogator at 4 kHz sampling, 1 m channel spacing, and 10 m gauge length.

Microseismic events induced by high-pressure fluid propagation were detected on the DAS fiber during the DAS-monitored fracturing stages in Figure 6b. This study uses data from the 15th and 25th fracturing stages (annotated S15, S25 in Figure 6b). The horizontal fiber section captures direct P- and S-wave arrivals from the induced events. The raw 4 kHz strain-rate data were downsampled to 1 kHz and bandpass-filtered to 30–120 Hz to isolate the dominant microseismic frequency band.

### 4.2. Denoising Results

**Figure 7:**
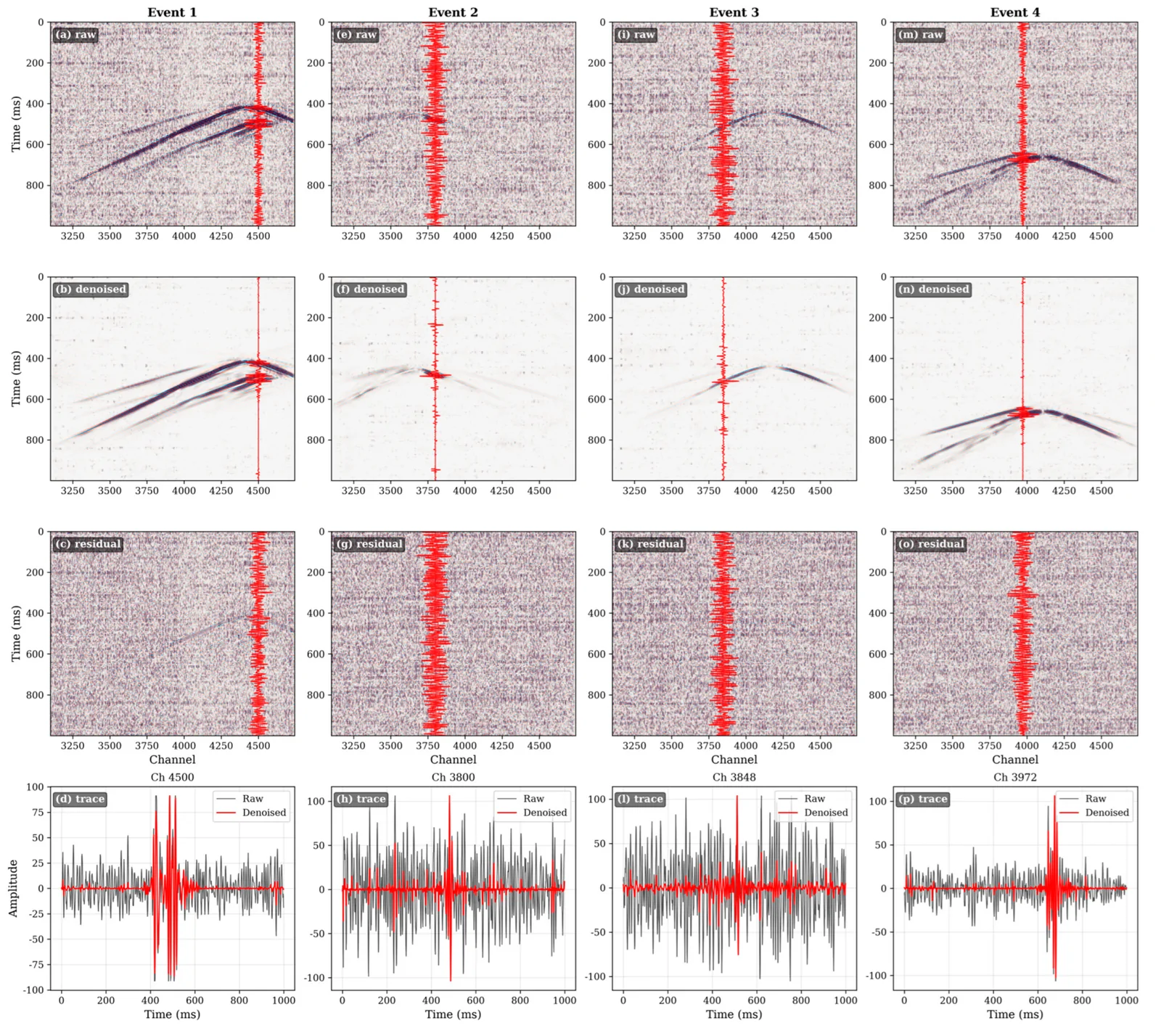
Curvelet MAD thresholding denoising results for four microseismic events (columns) from the Z1HF monitoring well. Rows show (top to bottom): raw record section, denoised output, residual (raw−denoised), and single-trace comparison at the channel with largest temporal standard deviation (red overlay). (**a**–**p**): Events 1–4 correspond to (**a**–**d**), (**e**–**h**), (**i**–**l**), and (**m**–**p**), respectively.

Curvelet MAD thresholding was applied to all cataloged microseismic events from the 15th and 25th fracturing stages, using the FDCT parameters ($N_{scales}=5,N_{angles}=32)$ and k=2.0. The denoising workflow follows the Methodology: forward FDCT, per-wedge MAD soft thresholding, and inverse FDCT. Figure 7 presents representative results for four microseismic events recorded on different dates during the treatment.

Event 1 (Figure 7a–d) contains a reflected phase in addition to the direct P and S arrivals. The raw record (Figure 7a) is strongly contaminated by the stochastic DAS noise floor, and after curvelet MAD denoising (Figure 7b) the P-wave, S-wave, and reflected phases all emerge as clearly visible, coherent wavefronts. The residual (Figure 7c) is dominated by spatially and temporally uncorrelated random noise; a slight trace of the reflected phase remains in the residual, but its magnitude is small and does not affect the downstream analysis. The recovery of this reflected phase is the point of denoising Event 1; DAS-recorded microseismic reflections image the induced fractures and faults that the direct arrivals alone cannot resolve [5,6,7], and a reflected phase visible above the noise floor—rather than buried in it—is a prerequisite for any subsequent fracture-imaging workflow. Events 2 and 3 (Figure 7e–l) are weak-signal events; in the raw records the P-wave phase is buried in the noise and essentially invisible, while after denoising (Figure 7f,j) the P-wave becomes apparent and the S-wave phase stands out more clearly. Event 4 (Figure 7m–p) is similar: after denoising, both the P- and S-wave phases are markedly clearer than in the raw record.

Across these events the practical payoff of denoising is that the P-wave—which is buried in the noise on the raw records and essentially unusable—becomes pickable; recovering an independent P arrival in addition to the S arrival adds a complementary travel-time constraint, so that subsequent joint P–S inversion and event location are better constrained than with the S arrival alone [1,2]. In every event the residual panels confirm that the removed energy is incoherent stochastic noise with no coherent signal leakage at the S-wave onset, and the single-trace comparisons (Figure 7d,h,l,p) overlay the denoised trace (red) on the raw trace (black) at one representative channel; the random noise is markedly suppressed, while the onset and first-arrival characteristics we care about for picking—the sharp take-off and polarity of the P and S pulses—are clearly preserved.

**Figure 8:**
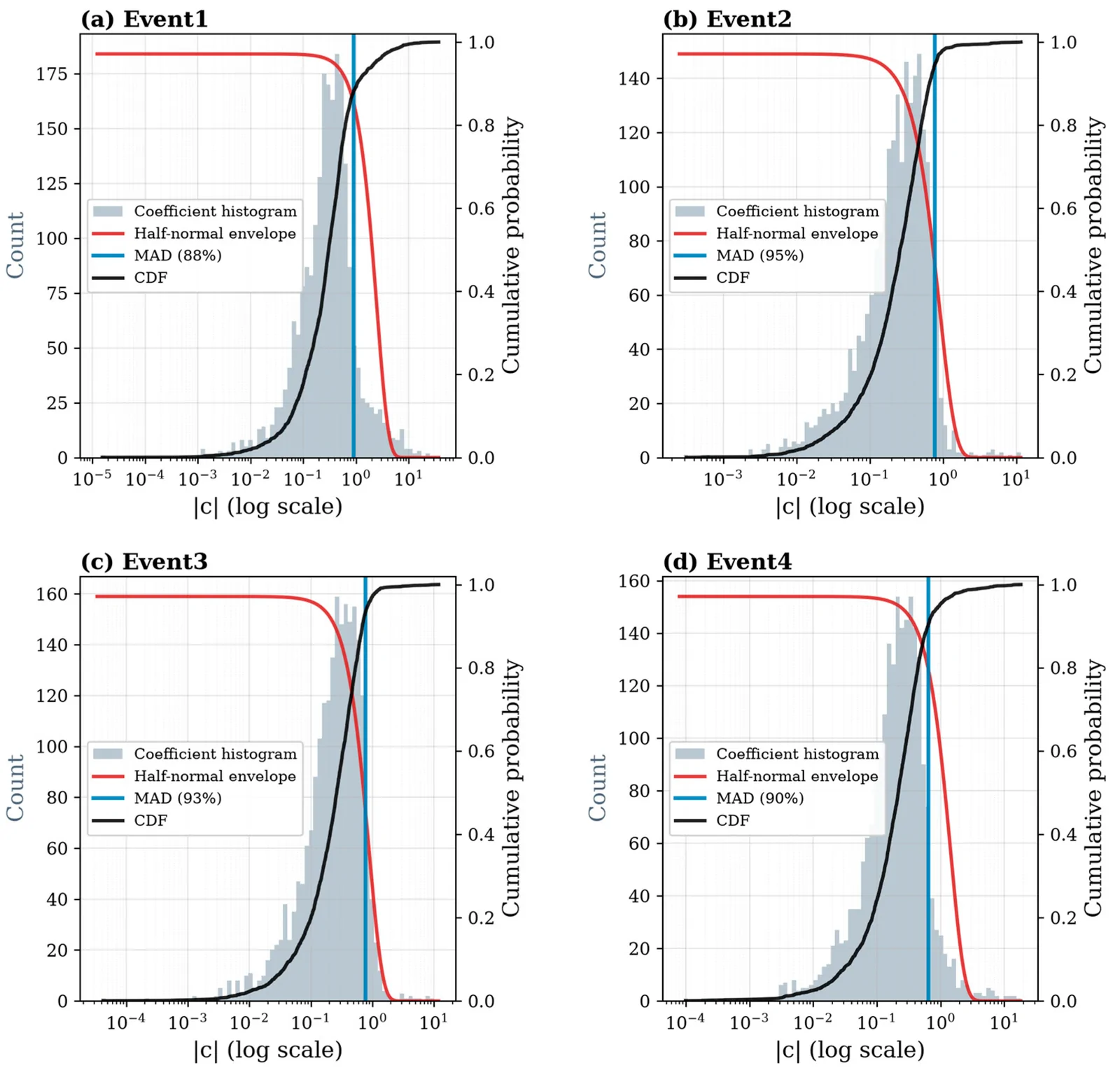
The coefficient-amplitude distribution of the most energetic wedge at scale 3 for four field events. (**a**–**d**) Events 1–4. Panels display the histogram (gray), half-normal fit (red), empirical CDF (black, right-hand axis), and the MAD threshold at default k=2.0 (blue vertical line), with the corresponding percentile position on the CDF annotated.

To verify whether the coefficient-level mechanism derived from synthetic data (Figure 5b) holds for these field records, we perform the same wedge-distribution analysis on four field events. We adopt the identical FDCT configuration used in our denoising workflow (Figure 8). For each event, amplitudes of the highest-energy wedge at scale 3 closely follow a half-normal distribution. This observation suggests that stochastic noise from the HiFi DAS interrogator remains approximately Gaussian within the curvelet domain. For the four analyzed events, the MAD threshold with default k=2.0 lies between 88% and 95% of the empirical CDF. This range aligns with the 93% position observed on synthetic data (Figure 5b). The out-of-box mechanism validated on synthetic data therefore operates identically on field records. The threshold is positioned within the noise tail; most stochastic noise components are eliminated.

### 4.3. Quantitative Evaluation of Detection Performance

To go beyond purely visual evaluation, we quantify the practical benefits of denoising by examining its influence on automatic event-detection performance. Field data lack noise-free ground-truth waveforms, so waveform-domain quantitative metrics such as ∆SNR cannot be calculated. We therefore target the key practical goal of denoising, which is to recover weak microseismic events. The standard seismic method Short-Term Average/Long-Term Average (STA/LTA) is applied uniformly across all channels of the S15 and S25 stage records, both before and after denoising. An event is regarded as detected once the trigger fires, and Table 1 summarizes the corresponding statistics. Without denoising, the STA/LTA detector identifies 137 events for stage 15 and 152 events for stage 25. Following curvelet MAD denoising, these numbers rise to 190 and 181, respectively. Most of these newly uncovered events correspond to weak arrivals originally buried beneath noise, analogous to Event 2 shown in Figure 7.

## 5. Discussion

### 5.1. Comparison with Reference Methods and Physical Mechanism

To show that curvelet MAD is a viable denoiser for this wavefield class, which is not to rank methods against each other, we benchmark it against two reference methods spanning alternative paradigms: 2D wavelet thresholding (isotropic sparse transform) and the adaptive Goldstein FK filter (FK-amplitude-based coherent enhancement). Each is used in its standard configuration; the comparison is about whether curvelet denoises comparably and why, not which is best.

The 2D discrete wavelet transform decomposes d(x,t) into approximation and detail sub-bands through separable filtering, with only three fixed orientations (horizontal, vertical, and diagonal) regardless of content. This isotropy is the key limitation for curved wavefronts. We use Daubechies-4 with J=5 levels, soft thresholding, and a level-dependent VisuShrink rule estimated per sub-band [12], which accounts for the scale-dependent noise coloring introduced by the strain-to-strain-rate spatial finite difference.

This adaptive Goldstein FK filter scales each Fourier component of a sliding 32×32 window by ${\left|E(f,k)\right|}^{\alpha }$ with α=0.8 [11]:

(9) $$\tilde{E}(f,k)={\left|E(f,k)\right|}^{\alpha }E(f,k),\;\;\alpha \in [0,1].$$

so that locally energetic spectral components are amplified.

**Figure 9:**
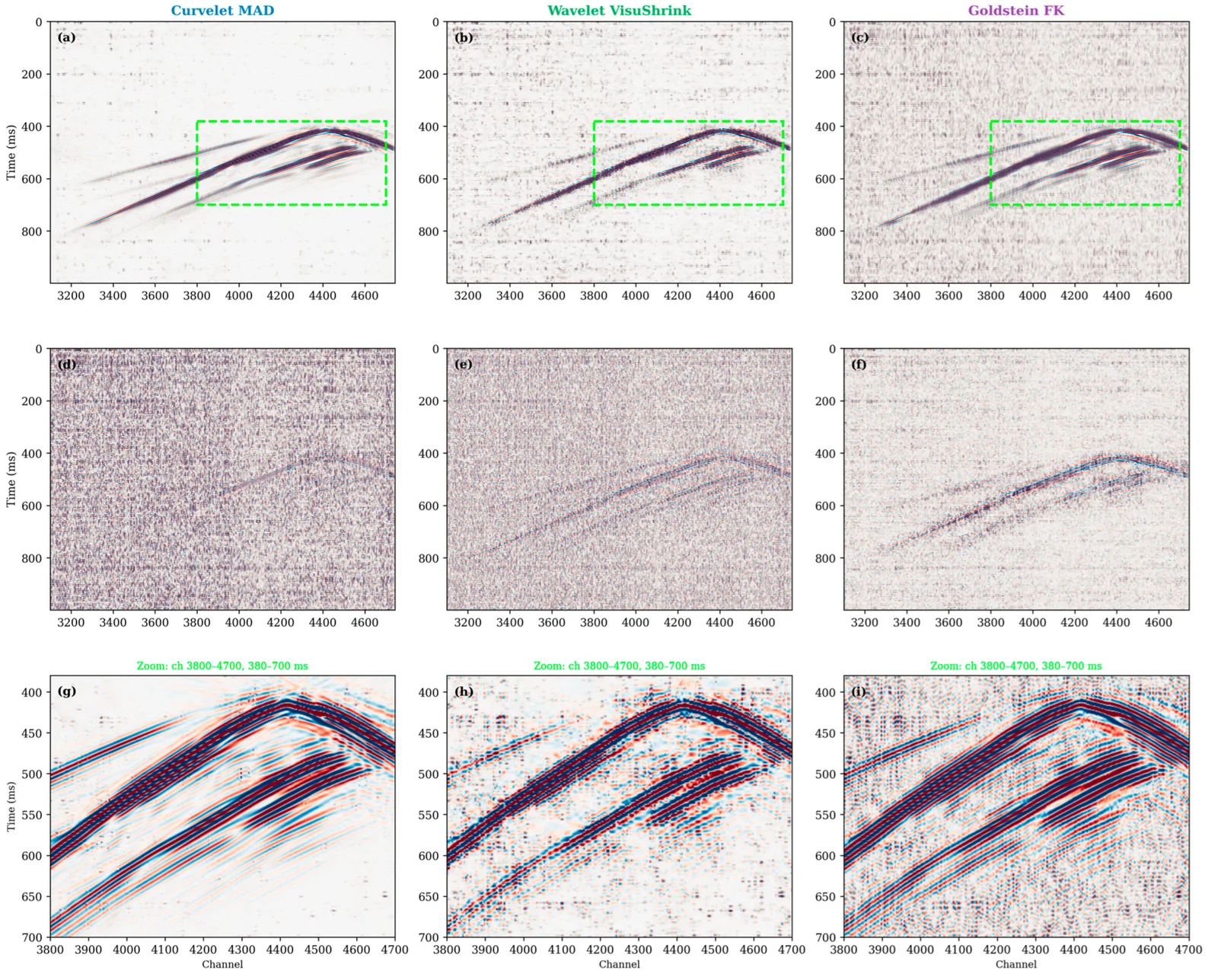
Three-method denoising comparison for Event 1. (**a**–**c**) Denoised outputs. (**d**–**f**) Residuals (raw minus denoised). (**g**–**i**) Zoomed views of the region indicated by the green box in (**a**).

**Figure A2:**
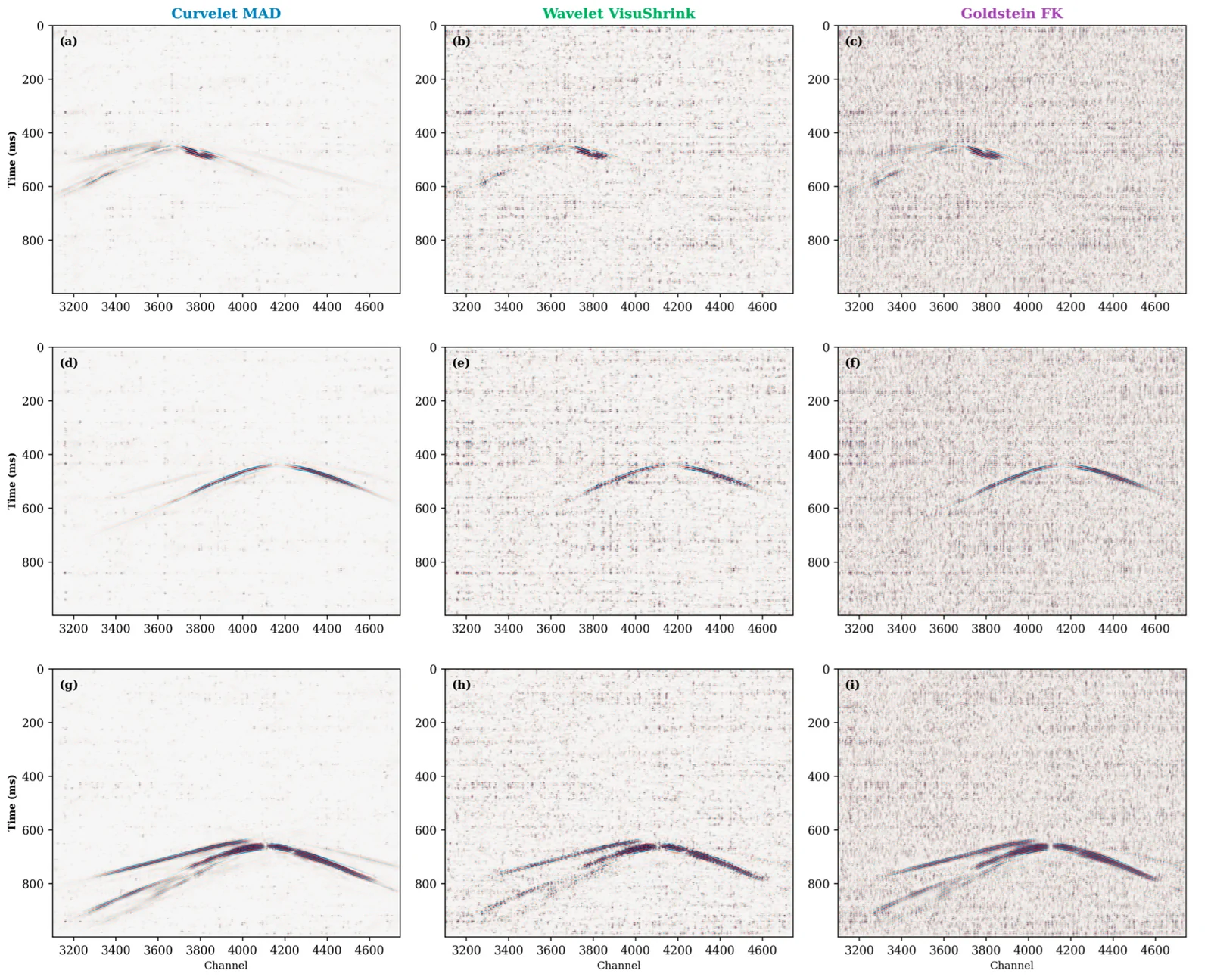
(**a**–**i**) Three-method denoising (curvelet MAD, wavelet VisuShrink, and Goldstein FK) applied to the other three events of Figure 7.

The three methods were applied to all four events of Figure 7. Figure 9 presents Event 1 (which contains a reflected phase) as a representative example; the results for the other three events are summarized in Appendix C (Figure A2).

Curvelet MAD (Figure 9a) recovers the S-wave and the reflected phase as continuous, coherent wavefronts with sharp onsets. Its residual (Figure 9d) contains only incoherent noise with no visible signal leakage, and the zoomed panel (Figure 9g) resolves the direct P-wave, S-wave, and reflected phase clearly. Wavelet VisuShrink (Figure 9b) also suppresses the background and recovers the principal arrivals, but the output is noticeably smoother, which reflected that phase is partially attenuated, faint coherent traces appear in the residual (Figure 9e), and the phase is less sharply defined in the zoom (Figure 9h). This behavior reflects the inherent limitation of an isotropic basis in representing curved wavefronts. The Goldstein FK filter (Figure 9c,f,i) provides meaningful noise suppression at a level broadly comparable to the wavelet result (Figure 9b,e,h). The STA/LTA detection statistics listed in Table 1 (Section 4.3), calculated from the continuous S15 and S25 stage records, offer quantitative support for the above visual comparison across all three methods. For S15, the wavelet and Goldstein FK outputs produce 166 and 179 detected events, while for S25 they yield 168 and 172, respectively. Both methods achieve substantial improvements relative to raw records, yet curvelet MAD delivers the most prominent performance gain.

We describe behavior of all three methods, not a ranking. Because each method is used only in its standard configuration, a well-tuned adaptive wavelet rule (e.g., SUREShrink/BayesShrink) could narrow the gap with curvelet, just as re-tuning could improve the Goldstein result. We make no claim of global optimality, nor do we argue for its superiority over tuned baseline algorithms. Our comparison only shows that the curvelet MAD approach, without per-dataset tuning, serves as an alternative option to remove random noise.

The relative behavior of the three methods follows from how well each frame matches the DAS microseismic wavefield. The $C^{2}$ wavefront geometry matches the curvelet’s design target of curvilinear discontinuities [25], whereas isotropic wavelet atoms need many coefficients to approximate a curved edge and so reduce the signal–noise separation; the directional energy concentration in the FK domain (Figure 1) aligns with the curvelet’s polar wedge partitioning but not with the Cartesian FK grid of the Goldstein filter. The extreme curvelet-domain energy concentration (see Figure 5) makes signal-bearing coefficients statistical outliers one to two orders of magnitude above the noise floor, so a per-wedge threshold in the noise tail naturally separates them.

The contribution of this comparison is the narrow one that curvelet MAD is a viable, mechanistically understood denoiser for the sparse-pulse regime of downhole DAS microseismic data, and that wavelet and Goldstein FK filtering are likewise viable options with their respective strengths (computational simplicity for wavelet; effectiveness on continuous, locally coherent signals for Goldstein).

### 5.2. Why a Sparse-Transform Approach Rather than Deep Learning

The choice of a sparse-transform approach over the self-supervised deep-learning denoising [17] is motivated by three considerations. One key merit lies in physical interpretability: curvelet thresholding is an explicit, per-wedge statistical operation, which means that the threshold is a quantity one can inspect, and the residual is a direct record of what was removed. In contrast, a trained network offers little mechanistic insight into its denoising behavior. Another practical advantage is transferability without dataset-specific training. Self-supervised networks must be trained on data from the specific deployment, and their performance degrades when interrogator hardware, fiber coupling, or acquisition geometry changes. The MAD threshold is estimated directly from each record and requires no training, consistent with the out-of-box philosophy of this study. Diagnosability forms the third consideration. Our denoising method is governed by a single transparent multiplier k, and its failure modes can be traced analytically, as demonstrated by our knee-point diagnosis. We nonetheless acknowledge that learned denoising methods hold a clear advantage for the non-Gaussian, non-stationary noise components excluded from our scope and view their combination with adaptive transform-domain thresholding as a promising direction.

### 5.3. Limitations and Future Work

Although curvelet soft thresholding coupled with MAD estimation represents a classic and well-established denoising framework [21], our work provides novel application-specific insights for downhole DAS microseismic data. Several boundary conditions and natural extensions are noted below.

Noise model. The additive white Gaussian noise model is a reasonable approximation for interrogator-dominated stochastic noise [27]. Two DAS-specific features—optical fading and temperature-induced baseline wander—introduce non-Gaussian, non-stationary components that the current spatially uniform per-wedge threshold does not explicitly model. The median-filter pre-processing step mitigates fading artifacts; adaptive thresholding represents a natural direction for handling time-varying noise conditions.

Scope of demonstration. The field deployment establishes single-well feasibility with one interrogator type. DAS noise characteristics depend on interrogator hardware, fiber coupling quality, and geological setting. The FDCT parameters (5 scales; 32 angles) are determined by data dimensions and the P/S resolution requirement and are expected to transfer across deployments. The threshold multiplier k=2.0 (a commonly used heuristic, [21]) proved effective on both synthetic and our tested field data without modification. Further validation across diverse wells, interrogator hardware, fiber-coupling scenarios and geological settings is required for broader deployment.

Future directions. The curvelet frame’s directional wedges are inherently suited to subsequent multi-phase processing (e.g., wavefield separation by apparent velocity); integrating denoising and wavefield decomposition within a single curvelet-domain workflow is a promising direction. The demonstrated directional sparsity is a necessary precondition for transform coding; whether it yields a practical DAS compression ratio is an open question we leave to future work.

## 6. Conclusions

This study has developed and evaluated a curvelet-transform framework for stochastic noise suppression in downhole DAS microseismic data. Using synthetic wavefields, we systematically compared four thresholding strategies at their standard default configurations and validated the recommended approach on continuous field recordings from a horizontal monitoring well in a shale gas field in southwest China.

Three strategies (MAD, quiet-window, and ECDF-percentile) perform reliably out of the box, with their default parameters generalizing well to the downhole DAS microseismic setting. The knee-point strategy, despite serving as the fallback default in the widely used DASPy toolbox, fails at its nominal parameter, for which the threshold lies inside the noise body and suppresses virtually nothing. Parameter sweeps demonstrate that this is a tuning issue rather than a fundamental weakness. At higher β, the knee-point strategy becomes competitive, but the outcome underscores the practical importance of well-chosen defaults.

Mechanistically, curvelet denoising is well suited to the curvilinear-wavefront geometry of DAS microseismic records. It produces denoised outputs with sharper signal onsets, more continuous wavefronts and cleaner residual noise. Therefore, curvelet MAD denoising stands as one viable option among competing processing methods.

## Figures and Tables

**Table 1:** Comparison of STA/LTA detection statistics before and after denoising for the S15 and S25 stages.

Stage	Raw	Curvelet MAD	Wavelet VisuShrink	Goldstein FK
15	137	190	166	179
25	152	181	168	172

## Data Availability

The datasets used and analyzed during the current study are available from the corresponding author upon request.

## Abbreviations

DAS	Distributed Acoustic Sensing
FDCT	Fast Discrete Curvelet Transform
MAD	Median Absolute Deviation
ECDF-percentile	Empirical Cumulative Distribution Function
CDF	Cumulative Distribution Function
FK	frequency–wavenumber

## Appendix A

For completeness, we state the hard thresholding operator referred to in the Denoising Framework. Given a threshold λ, hard thresholding zeroes coefficients whose absolute value falls below λ and leaves supra-threshold coefficients unchanged:

The corresponding soft thresholding operator is given by Equation (4) in the main text. Hard thresholding is an unbiased estimator—it preserves large coefficients exactly—but is discontinuous at ±λ. When applied in a transform domain whose basis functions are smooth and oscillatory (such as the curvelet frame), these discontinuities back-project into the spatial domain as low-amplitude ringing artifacts along wavefront edges [20]. Soft thresholding removes the discontinuity by additionally shrinking the surviving coefficients by λ, which makes the estimator continuous at the cost of a small bias. For the extreme-sparsity regime of DAS microseismic data in the curvelet domain—where signal coefficients are one to two orders of magnitude larger than the noise floor—this bias is negligible, and the continuity of soft thresholding yields visually cleaner reconstructions [21]. Figure A1 plots the two operators side by side.

## Appendix B

The algorithm proceeds in five steps.

Step 1: Source time function. The far-field particle velocity pulse follows the [30]:

(A1) $$s(t)=M_{0}\;\omega_{c}^{2}\;t\;e^{-\omega_{c}t},\;t\geq 0.$$

where $M_{0}=10^{1.5M_{w}+9.1}$ N·m [31] is the scalar seismic moment and $\omega_{c}=2\pi f_{c}$, with corner frequency $f_{c}\approx 150$ Hz for $M_{w}=0.05$. The pulse rises to a peak at $t=1/\omega_{c}$ and decays exponentially; its spectrum is flat below $f_{c}$ and decays as $f^{-2}$ above $f_{c}$, consistent with the $\omega^{-2}$ source model.

Step 2: Radiation pattern. The P and SV far-field radiation amplitudes $R_{P}^{\left(i\right)}$, $R_{SV}^{\left(i\right)}$, and $R_{SH}^{\left(i\right)}$ at receiver i are computed analytically from the source mechanism and the take-off angles [29].

Step 3: Geometric spreading and DAS axial projection. In a homogeneous whole-space, the far-field strain-rate amplitude factor for a body wave combines 1/r geometric spreading with an impedance-dependent $1/v^{2}$ factor (strain rate is the spatial derivative of particle velocity):

(A2) $$A_{P}^{\left(i\right)}=\frac{1}{4\pi r^{\left(i\right)}\rho v_{P}^{2}},\;\;A_{S}^{\left(i\right)}=\frac{1}{4\pi r^{\left(i\right)}\rho v_{S}^{2}}.$$

where ρ is density. DAS measures the projection of particle velocity onto the fiber axis $\hat{f}$ (uniaxial sensitivity; [32]). For a straight horizontal fiber deployed along the x-axis, $\hat{f}=\hat{x}$. The projection factors are given:

(A3) $$cos\theta_{P}^{\left(i\right)}=|{\hat{r}}_{i}\cdot \hat{x}|,\;\;cos\theta_{SV}^{\left(i\right)}=|{\hat{p}}_{SV}^{\left(i\right)}\cdot \hat{x}|,\;\;cos\theta_{SH}^{\left(i\right)}=|{\hat{p}}_{SH}^{\left(i\right)}\cdot \hat{x}|.$$

where ${\hat{r}}_{i}$ is the unit ray vector from source to receiver (P-wave polarization), ${\hat{p}}_{SH}^{\left(i\right)}={\hat{r}}_{i}\times \hat{z}$ is the SH polarization, and ${\hat{p}}_{SV}^{\left(i\right)}={\hat{p}}_{SH}^{\left(i\right)}\times {\hat{r}}_{i}$ is the SV polarization.

A wave whose particle motion is orthogonal to the fiber produces zero DAS response regardless of its true amplitude. Figure 2c illustrates the projection geometry; for channels closest to the source, the P-wave ray is nearly orthogonal to the horizontal fiber, so $\left|cos\theta_{P}\right|$ is small and P-wave amplitudes are strongly suppressed; S-wave particle motion has larger fiber-parallel components, yielding larger projection factors.

Step 4: Travel times. P- and S-wave travel times $t_{P}^{\left(i\right)}=r^{\left(i\right)}/v_{P}$ and $t_{S}^{\left(i\right)}=r^{\left(i\right)}/v_{S}$ are computed from the straight-line source–receiver distance $r^{\left(i\right)}$.

Step 5: Superposition. The DAS record for channel i is:

(A4) $$\begin{array}{c} d_{i}(t)=R_{P}^{\left(i\right)}cos\theta_{P}^{\left(i\right)}A_{P}^{\left(i\right)}\;s(t-t_{P}^{\left(i\right)})\\ +\left[R_{SV}^{\left(i\right)}cos\theta_{SV}^{\left(i\right)}+R_{SH}^{\left(i\right)}cos\theta_{SH}^{\left(i\right)}\right]A_{S}^{\left(i\right)}\;s(t-t_{S}^{\left(i\right)}). \end{array}$$

The source wavelet s(t) is scaled by the combined amplitude factor, shifted by linear interpolation to the arrival time, and summed across wave types.

## Appendix C

To confirm that the behavior observed in Figure 9 is consistent rather than specific to Event 1, Figure A2 applies all three methods to the other three events as Figure 7.
